# TBXAS1 Gene Polymorphism Is Associated with the Risk of Ischemic Stroke of Metabolic Syndrome in a Chinese Han Population

**DOI:** 10.1155/2022/9717510

**Published:** 2022-07-25

**Authors:** Jie Peng, Fanghong Lu, Ming Zhong, Yingxin Zhao, Zhihao Wang, Wei Zhang

**Affiliations:** ^1^The Key Laboratory of Cardiovascular Remodeling and Function Research, Chinese Ministry of Education, Chinese National Health Commission and Chinese Academy of Medical Sciences, the State and Shandong Province Joint Key Laboratory of Translational Cardiovascular Medicine, Qilu Hospital of Shandong University, Jinan, Shandong, China; ^2^Department of Geriatric Medicine, Qilu Hospital of Shandong University, Key Laboratory of Cardiovascular Proteomics of Shandong Province, Jinan, China; ^3^Cardio-Cerebrovascular Control and Research Center, Institute of Basic Medicine, Shandong Academy of Medical Sciences, Jinan, China

## Abstract

**Objective:**

To investigate the association between thromboxane A synthase 1 (*TBXAS1*) gene polymorphism and metabolic syndrome (MS) and explore whether gene polymorphism could act as biomarkers in MS and its components or whether it could play a role in MS-related damage.

**Methods:**

A total of 3072 eligible subjects were obtained, of which 1079 cases were controls and 1993 cases were MS patients. Subjects were followed up for 5 years, and the endpoint were recorded. The gene polymorphism of *TBXAS1* was detected by using the Sequenom MassArray method.

**Results:**

Significant differences were observed in ischemic stroke and NC_000007.14: g.139985896C>T (*P* < 0.05). The incidence of ischemic stroke was significantly higher in T allele carriers than in C (*P* < 0.05). C allele was the protective factor of the onset of ischemic stroke. There were negative interactions between C allele and waist circumference (WC), systolic blood pressure (SBP), diastolic blood pressure (DBP), triglycerides (TG), high-density lipoprotein cholesterol (HDL-C), and fasting plasma glucose (FPG).

**Conclusion:**

These findings suggest that NC_000007.14: g.139985896C>T was related to the incidence of ischemic stroke in the whole and MS population, and individuals who carry the C allele have a reduced risk of ischemic stroke, which may be used as a promising biomarker of disease risk in patients with MS.

## 1. Introduction

Metabolic syndrome (MS) is a clustering of some cardiovascular risk factors, including obesity, hypertension, hyperglycemia and dyslipidemia. In recent years, with the effects of an aging population, overeating, lack of exercise and other adverse lifestyles, the prevalence of MS has continued to increase globally. Epidemiological studies in western countries showed that, the prevalence of MS in adults is about 20-30% [1]. In China, a survey showed that the prevalence of MS was approximately 18%-22% [2]. As a chronic lifelong disease, MS is closely related to diabetes and cardiovascular disease. Previous studies have confirmed that MS can increase the risk of diabetes by 5 times [3], increased the risk of cardiovascular events by 2 times [4], and increased all-cause mortality by 1.5 times [5]. In addition, MS is an important risk factor for stroke, which could increase the risk of ischemic stroke by a factor of 2 [6]. Therefore, it is necessary to study the MS and related diseases.

Studies have shown that [7], multiple risk factors are related to the onset and prognosis of MS, but even when exposed to the same environment, some populations are still more prone to MS or target organ damage than others. Recent studies have found MS has family aggregation, as well as racial and regional differences [8, 9]. At the same time, the components of MS, such as hypertension, abnormal glucose and lipid metabolism, also have a genetic predisposition [10, 11], which indicate that MS has a significant genetic susceptibility. Therefore, it is currently believed that MS is a complex disease with the combined action of genetic factor and environmental factor, in which genetic factor is internal factor that play an important role in the development of MS. In recent years, several susceptibility genes related to MS and its components have been identified, such as the retinol-binding protein 4 gene [12, 13], angiopoietin-like protein gene [14], and glucokinase regulatory protein gene [15]. These genes mainly affect the onset and prognosis of MS by affecting the intermediate phenotypes of MS, therefore, by studying the susceptibility genes related to MS, we can not only elucidate the pathogenesis of the disease from the genetic perspective, but also screen the susceptible population at an early stage, providing new ideas and prospects for the comprehensive treatment of MS.

Thromboxane synthase (TS) is a microsomal enzyme that belongs to the cytochrome P450 (CYP450) family. TS is listed as CYP5 according to the nomenclature of P450 [16, 17]. The function of TS is to catalyze the isomerization of prostaglandin H2 (PGH2) to thromboxane A2 (TXA2). TXA2 is an effective inducer of platelet aggregation, release and smooth muscle contraction, playing an important role in regulating vascular tension, maintaining blood fluidity and hemostatic mechanism [18]. Lack of platelet TS activity can lead to bleeding, while excess of TXA2 is associated with the pathological process of many diseases, such as cardiovascular diseases [19]. Thromboxane A synthase 1(*TBXAS1*) gene is located on chromosome 7q34 [20, 21], including 13 exons and 12 introns [21, 22]. As its important role in thrombotic diseases, it has become a research focus in recent years. Oh et al. [23] reported that the rare allele of NC_000007.14:g.139971318T>A may play a protective role against aspirin hypersensitivity via a lower catalytic activity of the *TBXAS1* gene. Lemaitre et al. [24] studied more than 30 single nucleotide polymorphisms (SNPs) in the *TBXAS1* gene and found that NC_000007.14:g.139954457C>T, NC_000007.14:g.139985896C>T, and NC_000007.14:g.139964799A>C are all related to the onset of myocardial infarction, and NC_000007.14:g.139985896C>T is also associated with ischemic stroke. As the risks of cardiovascular and cerebrovascular disease in MS patients are significantly higher than that in normal people, and the relationship between this gene and MS has not been reported, Whether *TBXAS1* gene is a susceptible gene for MS and its components, or plays a role in MS-related damage needs further exploration and verification.

Previous studies have shown that, the release of inflammatory mediators is an important factor in the inflammatory disease models, which play a role as biomarkers in the occurrence and development of inflammatory diseases. Isola et al. [25] analyzed the association among nod-like receptor family pyrin domain-containing protein-3 (NLRP3) levels in patients with periodontitis and type-II diabetes mellitus (DM) and demonstrated that NLRP3 was a promising biomarker of disease risk. Increased transglutaminase2 (TG2) from periodontitis patients could be associated with high levels of pro-inflammatory markers promoting the interaction between molecular mechanisms involved in bone remodeling and resorption [26]. Recent study assessed the prevalence of the periodontal biotype in a group of patients and found that female subjects presented a higher prevalence of thin gingival biotype respect to male subjects and that no relationship was found between gingival biotypes and malocclusion [27]. Based on these findings, we consider that MS is a multifactorial disease, which has association with inflammation, gene polymorphisms may act as biomarkers in this disease. Moreover, there were few reports studied this interesting topic with this kind of study design.

For the above reasons, the purposes of this cohort study are to examine distribution of *TBXAS1* gene polymorphism in this population, investigate the associations between *TBXAS1* gene polymorphism and MS, and explore whether gene polymorphism could act as biomarkers in MS and its components, or plays a role in MS-related damage, then ultimately optimizing the preventive and treatment strategies.

## 2. Materials and Methods

### 2.1. Study Population

This is a cohort study. Participants were the Han population from Shandong Province, China, surveyed from January 2007 to December 2007. The sample size of this study was determined based on the prevalence of MS. By numbering and random sampling, several rural counties and city region were randomly selected, the communities (around 500 to 1,000 households each) from the chosen county and city were randomly selected, respectively. Eventually, 21,700 people were surveyed and 3,072 subjects met the requirements, of which 1,079 cases were normal controls and 1,993 cases were patients with MS. From January 2012 to December 2012, the study subjects were followed up and the endpoint events were recorded.

This study was approved by the ethics committee of Shandong Academy of Medical Sciences. We followed the ethical guidelines of the declaration of Helsinki, and written informed consent was obtained from all participants.

### 2.2. Inclusion and Exclusion Criteria

Participants who met these criteria were eligible for inclusion: (1) aged 18 to 75 years; (2) MS group: with MS. The definition of MS required at least three of the following components: (a) abdominal obesity (waist circumference (WC) ≥ 90 cm in Chinese men and ≥80 cm in Chinese women; (b) elevated blood pressure: systolic blood pressure (SBP) ≥ 130 mm Hg, diastolic blood pressure (DBP) ≥ 85 mm Hg, or known treatment for hypertension; (c) elevated triglycerides (TG): fasting plasma TG ≥ 150 mg/dL (1.7 mmol/L), drug treatment for elevated TG was an alternate indicator; (d) low high-density lipoprotein cholesterol (HDL-C): fasting HDL − C < 1.0 mmol/L in men and <1.3 mmol/L in women, drug treatment for reduced HDL-C was an alternate indicator; and (e) hyperglycemia: fasting plasma glucose (FPG) level of ≥5.6 mmol/L (≥100 mg/dL) or known treatment for diabetes, and drug treatment of elevated glucose was an alternate indicator [28]. And lastly, (3) control group: normal, healthy participants.

All the subjects meeting the exclusion criteria below could not be included in the study: with secondary hypertension, severe heart failure, renal failure, or valvular heart disease. Individuals with missing covariates, missing biochemical data, or undetected or discordant genotype were also excluded. Endpoint is ischemic stroke.

### 2.3. Demographic Data Collection, Clinical and Biological Assessment

The clinical data were assessed by questionnaire. Height, weight and WC were measured and body mass index (BMI) was calculated. Blood pressure was measured with the use of OMRON HEM-7011 electronic sphygmomanometer (Omron, Japan), SBP and DBP were defined as the average of three readings. Venous blood samples were obtained from all subjects after fasting for at least 12 hours for biochemical determination and DNA extraction.

### 2.4. SNPs Selection and Genotyping

SNPs were selected based on the following criteria. Firstly, SNPs located within genes with a MAF of >5% in CHB according to the NCBI HapMap Database (http://hapmap.ncbi.nlm.nih.gov/).Secondly,selected SNPs were entered into Haploview Ver. 4.2 software [29] to obtain tag SNPs. Thirdly, no studies have addressed the distribution regularities of the SNPs, or their relationship with this study population. A total of 29 SNPs that may be related to the characteristics of the population were chosen, according to the preliminary screening results of the previous data and reports. At last, the NC_000007.14:g.139985896C>T of *TBXAS1* gene was chosen and further statistical analysis was performed.

Genomic DNAs were extracted from blood by Magen blood DNA kit D3133-03 (Magen, Guangzhou, China) following the manufacturer's protocols. SNP at NC_000007.14:g.139985896C>T was genotyped in BGI, Shenzhen, China by Sequenom MassArray system (Sequenom, San Diego, CA). The PCR reaction was conducted using GeneAmp PCR System 9700 (ABI, Foster City, CA, USA). And mass determination was performed with matrix-assisted laser desorption ionization time-of-flight (MALDI-TOF) mass spectrometry. Data were collected by Spectro TYPER Ver. 4.0 software (Sequenom, San Diego, CA). Call rates of genotyping were >95% for the SNP. A total of 120 (5%) samples were randomly selected for the concordance test, and the concordance rates were >99% for the SNP [30].

### 2.5. Statistical Analysis

Statistical analysis was conducted with SPSS Ver. 17.0 (SPSS, Chicago, IL). Continuous variables were presented as mean and SD or SEM, and compared by Student's *t* test, paired *t* test or analysis of variance (ANOVA) with post hoc least-significant differences *t* test. Categorical variables were presented as percentages, and compared by *χ*^2^ test. Multivariable Logistic regression was used to analyze the relationship between SNPs and endpoint events in different populations; the degree of association was expressed by odds ratio (OR) and 95% confidence interval (CI). The interaction between gene polymorphism and risk factors was substituted into the equation. Hardy-Weinberg equilibrium for NC_000007.14:g.139985896C>T was tested using *χ*^2^ goodness-of-fit test. *P* values were two-tailed and considered significant when less than 0.05.

## 3. Results

### 3.1. Characteristics of the Study Population

**Figure 1:**
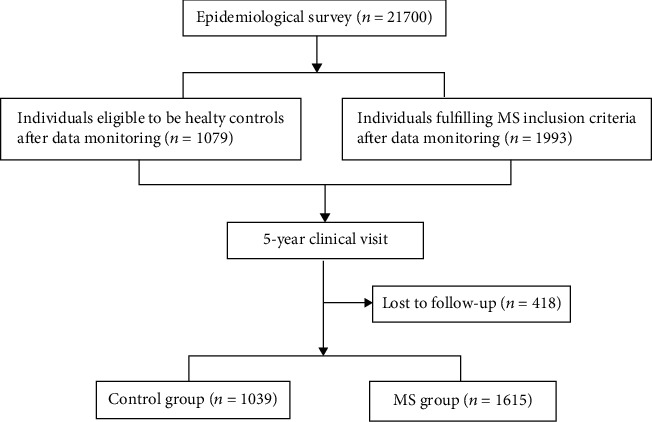
Disposition of the subject.

The baseline characteristics of the subjects were shown in Table 1. There were 1993 cases in the MS group and 1,079 cases in the control group. The sex and age of the control group and the MS group could be matched. During the 5-year follow-up, 418 subjects were lost to follow-up; 1,615 patients in the MS group and 1,039 patients in the control group were completed (Figure 1); and a total of 37 endpoints occurred: 30 in the MS group and 7 in the control group.

Genotype analysis was performed on NC_000007.14:g.139985896C>T of the 3072 samples. Finally, there were 1,991 cases in the MS group and 1,076 cases in the control group completed. The frequency distribution of NC_000007.14:g.139985896C>T genotypes and alleles in control group and MS group was basically consistent with that in HAPMAP-CHB; the frequency distribution of NC_000007.14:g.139985896C>T in both groups did not deviate from the Hardy-Weinberg equilibrium.

Table 2 presents the genotype and allele frequencies of the NC_000007.14:g.139985896C>T in the MS group and controls. There were no significant differences in the NC_000007.14:g.139985896C>T genotype or the allele distributions between MS and controls (*P* > 0.05). The comparison of baseline clinical characteristics between different genotypes in the control group and the MS group showed that there was no significant difference in clinical indicators between different genotypes in the MS group (*P* > 0.05). But significant difference was observed in the control group, compared with CC genotype carriers, the CT genotype carriers had significantly higher low-density lipoprotein cholesterol (LDL-C) (*P* < 0.05). (Table 3).

### 3.2. Relevance of SNP and Components of MS

The components of MS include TG, blood pressure, FPG, abdominal obesity, HDL-C, the relationship between the NC_000007.14:g.139985896C>T genotypes and the components were analyzed (Table 4). The results showed that there was no significant difference between the groups according to TG, blood pressure, FPG, and abdominal obesity (*P* > 0.05). It suggested that there was no association between genotypes and these components. But in group of HDL-C, significant differences were observed in the distributions of the CC genotype and the C allele of NC_000007.14:g.139985896C>T between normal HDL-C group and abnormal HDL-C group (*P* = 0.023 and *P* = 0.018, respectively). The HDL-C abnormality of the C allele is significantly higher than the T allele carriers (*P* < 0.05).

### 3.3. Relevance of SNP and Endpoint

The relationship between the NC_000007.14:g.139985896C>T genotypes and the incidence of ischemic stroke was analyzed of the whole population, MS group and controls. The results showed that there was significant difference in the whole population and MS group (*P* < 0.05) (Table 5). The incidence of ischemic stroke was significantly higher in T allele carriers than in C allele carriers (*P* < 0.05). In the control group, no endpoint was associated with this SNP (*P* > 0.05).

### 3.4. Risk Factors of the Onset of Ischemic Stroke

The Logistic regression analysis was performed for screening the risk factors of the onset of ischemic stroke (Table 6). The results showed that, in the whole population, C allele and gender was the protective factors of the onset of ischemic stroke, the risk of ischemic stroke was decreased when the individual was women or with C allele. SBP was the risk factor for the onset of ischemic stroke, the risk of ischemic stroke was increased when the SBP was higher; in the MS population, C allele and gender was the protective factors of the onset of ischemic stroke, the risk of ischemic stroke was decreased when the individual was women or with C allele.

The interaction effects between C allele and the components of MS on the onset of ischemic stroke showed that, in the whole population, there were negative interactions between C allele and WC, SBP, DBP, TG, HDL-C, and FPG; in the MS group, there were also negative interactions between C allele and WC, SBP, DBP, TG, HDL-C, and FPG. In the case of same WC, SBP, DBP, TG, HDL-C, and FPG, the onset of ischemic stroke of C allele carrier was reduced (Table 7).

## 4. Discussion

The current researches on candidate genes for MS are mainly focused on genes related to lipid metabolism disorders, hypertension, and so on. When these genes related to different components of MS have polymorphisms or mutations, they may touch some risk factors or protective factors of MS, thereby affecting the development of MS. In the early years, researches on *TBXAS1* gene mainly focused on inflammation, breast cancer, and other diseases. In recent years, researches on this gene and diseases such as myocardial infarction, stroke, and preeclampsia have become a hot spot. Ulrich et al. [31] found that mutations in the *TBXAS1* gene could alter protein function, which were related to inflammation and angiogenesis. Abraham et al. [32] found that the NC_000007.14:g.139959792T>A mutation of the *TBXAS1* gene could moderately increase the risk of breast cancer. Lemaitre et al. [24] found NC_000007.14:g.139959792T>A was also related to ischemic stroke. Park et al. [33] found that the +16184G> T polymorphism of *TBXAS1* gene and *TBXAS1-ht3* polymorphism frequency in cerebral infarction, especially in patients with small arterial occlusive cerebral infarction, increased significantly.

In our study, there was polymorphism in the NC_000007.14:g.139985896C>T in the Han population in Shandong province of China [34]. A comparison of the genotype and allele frequency distribution of the population suggested that there was no association between MS and the different genotypes of NC_000007.14:g.139985896C>T. However, MS is a syndrome of abdominal obesity, hyperglycemia, dyslipidemia, and hypertension. This study observed and recorded the main clinical indicators related to the components of the MS. The results showed that in the MS population, there was no correlation between NC_000007.14:g.139985896C>T polymorphism and clinical indicators. This study further studied the genotype and allele frequencies and the different components of MS. The results showed that this gene polymorphism had no relationship with TG, blood pressure, FPG, abdominal obesity, and HDL-C, suggesting that NC_000007.14:g.139985896C>T polymorphism has no correlation with the incidence of MS. But although there was no difference in HDL-C levels among different genotype carriers, there were differences in HDL-C levels between the C and T alleles, suggesting that the C allele may have a regulatory effect on HDL-C. Therefore, is NC_000007.14:g.139985896C>T related to the prognosis of MS? To this end, we followed the population for 5 years to observe the relationship between the gene polymorphism and the prognosis of MS. This study found that NC_000007.14:g.139985896C>T polymorphism in the general population and MS population is associated with the incidence of ischemic stroke, which was similar to Lemaitre's results that NC_000007.14:g.139985896C>T polymorphism is associated with ischemic stroke [24].

Recent studies also found that, *TBXAS1* gene polymorphisms had significant association with large-artery atherosclerosis stroke susceptibility and the level of *TBXAS1* expression [35]. A study of Chinese people found that the TT genotype of *TBXAS1* and T allele of NC_000007.14:g.139845571 T > G increase susceptibility to ischemic stroke [36]. However, the population of our study was more special, which were MS patients. At the same time, through screening of NC_000007.14:g.139985896C>T polymorphism and risk factors for ischemic stroke, it was found that the C allele was an independent protective factor for the incidence of ischemic stroke in the whole and MS population, individuals who carry the C allele have a reduced risk of stroke. Carriers of T allele are more likely to have ischemic stroke, but that was not an independent risk factor for ischemic stroke in this study, which may be influenced by other factors and need further study. Therefore, people carrying the C allele are less likely to suffer from ischemic stroke, which is more obvious when combined with MS. Patients with T allele should be better treated for MS to prevent the occurrence and development of ischemic stroke.

The components of MS, such as hypertension, glucose and lipid metabolism, are genetically predisposed [10, 11]. The heterogeneity of clinical phenotypes suggests that there may be genetic heterogeneity in MS, but no candidate genes have been reported could directly explain the onset and prognosis of MS. These genes mainly affect the occurrence and development of MS through the intermediate phenotype of MS development. Therefore, in this study, interaction analysis was performed of the NC_000007.14:g.139985896C>T polymorphism and the clinical characteristics of the subjects. Obesity is a risk factor of stroke [36], the results of this study showed that there were negative interactions between C allele and WC in the whole and MS population, suggesting that the C allele is a protective factor of ischemic stroke. With constant WC, the risk of ischemic stroke was reduced in carriers of the C allele. Hypertension is also a risk factor for stroke [37]. In the present study, with the genotype of the whole and MS population unchanged, those with high SBP or DBP have a high risk of stroke, which is consistent with previous researches. Abnormal glucose and lipid metabolism are the main clinical feature of MS. In this study, the C allele had negative interactions with TG, HDL-C, and FPG. With the same TG, HDL-C, and FPG, the C allele carriers had a reduced risk of ischemic stroke. It is worth noting that the interaction of genes and clinical characteristics refers to combined effect, and cannot simply add the effects of various factors together. At the same time, a distinction should be made between biologically relevant and statistically relevant differences. Therefore, the statistical interactions found by association analysis need further study of its molecular mechanism to clarify its biological significance.

*TBXAS1* gene encodes TS, which catalyze the isomerization of PGH2 to TXA2. TXA2 is a vasoconstrictor and inducer of platelet aggregation. The enzyme plays a role in several pathophysiological processes including hemostasis, cardiovascular disease, and stroke. NC_000007.14:g.139985896C>T is a intron polymorphism of *TBXAS1* gene. Although introns not encoded amino acid, it plays an important role in regulating gene expression, introns not only participate in precursor RNA splicing stage, but also involved in RNA transcription, editing, the nuclear transport, as well as the regulation of gene expression in the process of the decay rule of nonsense. Introns have bidirectional regulation of gene expression. Studies have shown that despite the promoter is intact, the gene still unexpressed when intron is lacking, which proves the importance of introns in gene expression [38]. The regulation of introns on gene expression is influenced by their internal SNPs [39–41], which often change mRNA levels by affecting transcription, RNA extension, splicing or maturation [42]. In this study, the C allele in this SNP polymorphism may interfere with the expression of *TBXAS1* and reduce the generation of TS, thereby reducing the occurrence of thrombotic diseases. According to the results of this study, in the whole and MS population, the subjects without ischemic stroke carried more C alleles, and the difference was statistically significant, which further demonstrated the protective effect of the C allele, and this biological function process needs to be further verified.

In recent years, exploring biomarkers that can early predict and diagnose diseases has become a research hotspot. Previous studies have shown that, NLRP3 was a promising biomarker of disease risk in patients with type-II DM [25], increased TG2 from periodontitis patients was associated with high levels of pro-inflammatory markers promoting the interaction between molecular mechanisms involved in bone remodeling and resorption [26]. Some studies also showed that, mutations in the *TBXAS1* gene could alter protein function, which were related to inflammation and angiogenesis [31], and *TBXAS1* gene polymorphisms had significant association with large-artery atherosclerosis stroke susceptibility [35]. In the present study, results demonstrate that *TBXAS1* gene NC_000007.14:g.139985896C>T polymorphism was related to the incidence of ischemic stroke in patients with MS. As MS is an important risk factor for stroke [6], we infer this gene polymorphism may affect inflammation and arteriosclerosis through influencing inflammatory factors, and thereby influence the occurrence and progression of ischemic stroke in patients with MS, which may be used as a biomarker in MS-related damage. To the author's knowledge, there is little data that correlates gene polymorphism and MS patients as an early subclinical biomarker of disease risk. In this regard, the present study provides a new idea for early intervention of disease.

Some limitations in this study need to be considered. First of all, the hypothetical molecular mechanism that how NC_000007.14:g.139985896C>T polymorphism affected the onset of ischemic stroke was not addressed in the present study. Secondly, further validation is needed for the predictive value of this SNP as risk factor of MS components and pharmacogenetic indicator in independent cohorts. Finally, the mechanism of this gene polymorphism affecting the incidence of ischemic stroke in MS patients need further study.

In conclusion, this study has indicated that *TBXAS1* gene NC_000007.14:g.139985896C>T polymorphism was not associated with the pathogenesis or components of MS. However, it was related to the incidence of ischemic stroke in the whole and MS population, and individuals who carry the C allele of NC_000007.14:g.139985896C>T have a reduced risk of stroke. The above results suggest that this gene polymorphism has demonstrated a promising biomarker of disease risk in patients with MS and providing early and subclinical diagnosis of MS-related damage, which may be used as a basis for genotype-guided individualized medication.

## Figures and Tables

**Table 1:** Characteristics of the study subjects.

	Control group (*n* = 1079)	MS group (*n* = 1993)
Sex (M/F)	298/781	485/1508
Age (yr)	55.07 ± 8.83	55.48 ± 9.30
BMI (kg/m^2^)	21.92 ± 2.62	27.15 ± 3.38∗∗
WC (cm)	74.02 ± 5.91	90.23 ± 8.25∗∗
SBP (mmHg)	117.74 ± 9.91	150.66 ± 21.07∗∗
DBP (mmHg)	73.74 ± 6.37	89.77 ± 10.81∗∗
TC (mmol/L)	4.14 ± 0.54	4.78 ± 1.16∗∗
TG (mmol/L)	0.90 ± 0.30	2.23 ± 1.46∗∗
HDL-C (mmol/L)	1.61 ± 0.33	1.52 ± 0.58∗∗
LDL-C (mmol/L)	2.53 ± 0.54	3.26 ± 0.86∗∗
FPG (mmol/L)	4.58 ± 0.51	5.96 ± 2.10∗∗
Smoking (%)	14.7%	8.4%∗∗
Alcohol drinking (%)	11.2%	12.1%

BMI: body mass index; WC: waist circumference; SBP: systolic blood pressure; DBP: diastolic blood pressure; TC: total cholesterol; TG: triglycerides; HDL-C: high-density lipoprotein cholesterol; LDL-C: low-density lipoprotein cholesterol; FPG: fasting plasma glucose. Compared with the control group, ^∗^*P* < 0.05, ∗∗*P* < 0.001.

**Table 2:** Genotype and allele distributions in MS and controls.

	Number (%) of cases		*P*
	Control group (*n* = 1076) *n* (%)	MS group (*n* = 1991) *n* (%)	
Genotype			
CC	589 (55)	1075 (54)	
CT	419 (39)	771 (39)	0.603
TT	68 (6)	145 (7)	
Dominant effect			
CC	589 (55)	1075 (54)	0.704
CT+TT	487 (45)	916 (46)	
Recessive effect			
CC+CT	1008 (94)	1846 (93)	0.334
TT	68 (6)	145 (7)	
Allele			
C	1597 (74)	2921 (73)	0.428
T	555 (26)	1061 (27)	

“*n*” is the number of subjects.

**Table 3:** Characteristics of the study subjects of different genotypes.

	Control group (*n* = 1076)			MS group (*n* = 1991)		
	CC (*n* = 589)	CT (*n* = 419)	TT (*n* = 68)	CC (*n* = 1075)	CT (*n* = 771)	TT (*n* = 145)
Sex (M/F)	158/431	115/304	24/44	269/806	183/588	32/113
Age (yr)	54.90 ± 8.78	55.36 ± 8.98	54.44 ± 8.45	55.37 ± 9.29	55.65 ± 9.38	55.57 ± 8.78
BMI (kg/m^2^)	21.93 ± 2.70	21.98 ± 2.56	21.46 ± 2.24	27.18 ± 3.43	27.16 ± 3.33	26.83 ± 3.20
WC (cm)	74.01 ± 5.62	74.04 ± 6.20	73.86 ± 6.51	90.35 ± 8.27	90.10 ± 8.34	90.13 ± 7.76
SBP (mmHg)	117.29 ± 10.21	118.36 ± 9.61	117.75 ± 9.09	151.16 ± 21.00	150.22 ± 21.08	149.50 ± 21.68
DBP (mmHg)	73.61 ± 6.53	73.88 ± 6.21	74.02 ± 6.08	89.97 ± 10.93	89.71 ± 10.73	88.55 ± 10.42
TC (mmol/L)	4.11 ± 0.55	4.19 ± 0.52	4.11 ± 0.54	4.75 ± 1.18	4.82 ± 1.12	4.85 ± 1.23
TG (mmol/L)	0.89 ± 0.30	0.91 ± 0.31	0.90 ± 0.30	2.21 ± 1.48	2.26 ± 1.45	2.30 ± 1.36
HDL-C (mmol/L)	1.62 ± 0.35	1.60 ± 0.29	1.64 ± 0.33	1.50 ± 0.51	1.54 ± 0.68	1.59 ± 0.54
LDL-C (mmol/L)	2.49 ± 0.55	2.59 ± 0.51∗	2.47 ± 0.53	3.24 ± 0.81	3.28 ± 0.91	3.26 ± 0.87
FPG (mmol/L)	4.58 ± 0.50	4.55 ± 0.52	4.71 ± 0.50	5.95 ± 2.04	5.97 ± 2.11	6.06 ± 2.39

BMI: body mass index; WC: waist circumference; SBP: systolic blood pressure; DBP: diastolic blood pressure; TC: total cholesterol; TG: triglycerides; HDL-C: high-density lipoprotein cholesterol; LDL-C: low-density lipoprotein cholesterol; FPG: fasting plasma glucose. Compared with the CC control group, ^∗^*P* < 0.05.

**Table 4:** Relationship between genotype and allele distributions and components of MS.

	Normal TG (*n* = 691) *n* (%)	Elevated TG (*n* = 1300) *n* (%)	*P*	Normal BP (*n* = 124) *n* (%)	Elevated BP (*n* = 1867) *n* (%)	*P*	Normal FPG (*n* = 1053) *n* (%)	Elevated FPG (*n* = 938) *n* (%)	*P*	Normal weight (*n* = 195) *n* (%)	Abdominal obesity (*n* = 1796) *n* (%)	*P*	Normal HDL-C (*n* = 1317) *n* (%)	Abnormal HDL-C (*n* = 674) *n* (%)	*P*
Genotype															
CC	387 (56.0)	688 (52.9)		66 (53.2)	1009 (54.0)		567 (53.9)	508 (54.2)		104 (53.3)	971 (54.2)		687 (52.2)	388 (57.6)	
CT	259 (37.5)	512 (39.4)	0.351	49 (39.5)	722 (38.7)	0.989	408 (38.7)	363 (38.7)	0.972	76 (39.0)	695 (38.7)	0.965	527 (40.0)	244 (36.2)	0.060
TT	45 (6.5)	100 (7.7)		9 (7.3)	136 (7.3)		78 (7.4)	67 (7.1)		15 (7.7)	130 (7.1)		103 j(7.8)	42 (6.2)	
Dominant effect															
CC	387 (56.0)	688 (52.9)	0.202	66 (53.2)	1009 (54.0)	0.926	567 (53.9)	508 (54.2)	0.893	104 (53.3)	971 (54.2)	0.880	687 (52.2)	388 (57.6)	0.023
CT+TT	304 (44.0)	612 (47.1)		58 (46.8)	858 (46.0)		486 (46.1)	430 (45.8)		91 (46.7)	825 (45.8)		630 (47.8)	286 (42.4)	
Recessive effect															
CC+CT	646 (93.5)	1200 (92.3)	0.366	115 (92.7)	1731 (92.7)	1.000	975 (92.6)	871 (92.9)	0.863	180 (92.3)	1666 (92.9)	0.772	1214 (92.2)	632 (93.8)	0.203
TT	45 (6.5)	100 (7.7)		9 (7.3)	136 (7.3)		78 (7.4)	67 (7.1)		15 (7.7)	130 (7.1)		103 (7.8)	42 (6.2)	
Allele															
C	1033 (74.7)	1888 (72.6)	0.153	181 (73.0)	2740 (73.4)	0.941	1542 (73.2)	1379 (73.5)	0.858	284 (72.8)	2637 (73.4)	0.801	1901 (72.2)	1020 (75.7)	0.018
T	349 (25.3)	712 (27.4)		67 (27.0)	994 (26.6)		564 (26.8)	497 (26.5)		106 (27.2)	955 (26.6)		733 (27.8)	328 (24.3)	

BP: blood pressure; TG: triglycerides; HDL-C: high-density lipoprotein cholesterol; FPG: fasting plasma glucose; “*n*” is the number of subjects.

**Table 5:** Relationship between genotype and allele distribution and ischemic stroke in the population.

	Whole people			MS group			Control group		
	N (*n* = 3030) *n* (%)	Y (*n* = 37) *n* (%)	*P*	N (*n* = 1961) *n* (%)	Y (*n* = 30) *n* (%)	*P*	N (*n* = 1069) *n* (%)	Y (*n* = 7) *n* (%)	*P*
Genotype									
CC	1650 (54.5)	14 (37.8)		1065 (54.3)	10 (33.4)		585 (54.7)	4 (57.1)	
CT	1174 (38.7)	16 (43.2)	0.012	758 (38.7)	13 (43.3)	0.004	416 (38.9)	3 (42.9)	1.000
TT	206 (6.8)	7 (19.0)		138 (7.0)	7 (23.3)		68 (8.4)	0 (0)	
Dominant effect									
CC	1650 (54.5)	14 (37.8)	0.047	1065 (54.3)	10 (33.4)	0.026	585 (54.7)	4 (57.1)	1.000
CT+TT	1380 (45.5)	23 (62.2)		896 (45.7)	20 (66.6)		464 (45.3)	3 (42.9)	
Recessive effect									
CC+CT	2824 (93.2)	30 (81)	0.012	1823 (93.0)	23 (76.7)	0.005	1001 (91.6)	7 (100)	1.000
TT	206 (6.8)	7 (19.0)		138 (7.0)	7 (23.3)		68 (8.4)	0 (0)	
Allele									
C	4474 (73.8)	44 (59.5)	0.008	2888 (73.6)	33 (55.0)	0.003	1586 (74.2)	11 (78.6)	1.000
T	1586 (26.2)	30 (40.5)		1034 (26.4)	27 (45.0)		552 (25.8)	3 (21.4)	

Y: with ischemic stroke; N: without ischemic stroke; “*n*” is the number of subjects.

**Table 6:** The risk factors of the onset of ischemic stroke.

		*P*	OR	95% CI	
				Lower	Upper
Whole people	C allele	0.003	0.270	0.115	0.636
	T allele	0.485	1.291	0.630	2.644
	Sex	0.000	0.210	0.103	0.430
	SBP	0.024	1.023	1.003	1.044
MS group	C allele	0.005	0.261	0.103	0.661
	T allele	0.256	1.612	0.707	3.675
	Sex	0.013	0.330	0.137	0.793

SBP: systolic blood pressure.

**Table 7:** Interaction effect between C allele and the characteristics on the onset of ischemic stroke.

	Whole				MS			
			95% CI				95% CI	
	*P*	OR	Lower	Upper	*P*	OR	Lower	Upper
WC∗C	0.001	0.985	0.975	0.994	0.000	0.983	0.973	0.992
SBP∗C	0.012	0.992	0.986	0.998	0.002	0.991	0.985	0.997
DBP∗C	0.002	0.985	0.976	0.994	0.001	0.984	0.974	0.993
TG∗C	0.018	0.643	0.447	0.926	0.003	0.574	0.399	0.825
HDL-C∗C	0.002	0.481	0.304	0.761	0.001	0.435	0.271	0.697
FPG∗C	0.029	0.850	0.734	0.983	0.019	0.838	0.723	0.971

WC: waist circumference; SBP: systolic blood pressure; DBP: diastolic blood pressure; TG: triglycerides; HDL-C: high-density lipoprotein cholesterol; LDL-C: low-density lipoprotein cholesterol; FPG: fasting plasma glucose.

## Data Availability

All data generated or analyzed during this study are included in this published article. The exclusive access to the original data is preserved by JP and could be viewed under reasonable request.
